# Early low-dose hydrocortisone is associated with a reduced risk of bronchopulmonary dysplasia in infants born at less than 26 weeks' gestational age

**DOI:** 10.3389/fped.2025.1582881

**Published:** 2025-04-17

**Authors:** Fang Yao, Zhifeng Huang, Xueyu Chen, Chuanzhong Yang, Qiuping Li, Bingchun Lin

**Affiliations:** ^1^ The Second School of Clinical Medicine, Southern Medical University, Guangzhou, China; ^2^ Department of Neonatology, Shenzhen Maternity and Child Healthcare Hospital, Southern Medical University, Shenzhen, Guangdong Province, China; ^3^ Department of Newborn Care Center, Senior Department of Pediatrics, The Seventh Medical Center of PLA General Hospital, Beijing, China; ^4^ Shenzhen Key Laboratory of Maternal and Child Health and Diseases, Shenzhen, China

**Keywords:** bronchopulmonary dysplasia, hydrocortisone, extremely preterm infants, cortisol, low-dose

## Abstract

**Objective:**

To determine whether administering low-dose hydrocortisone early in treatment reduces the risk of bronchopulmonary dysplasia (BPD) in infants born before 26 weeks of gestation

**Study design:**

This retrospective case-control study compared the incidence of Grade II^+^ BPD between infants who received hydrocortisone treatment and those who did not. Propensity score matching was used to ensure comparability between the groups, with a 1:1 match ratio based on gestational age and birth weight.

**Results:**

A total of 66 infants were included in the study. Those who received early low-dose hydrocortisone demonstrated a significantly lower risk of Grade II^+^ BPD incidence (*p* = 0.024). Additionally, early administration of low-dose hydrocortisone was associated with a shorter duration of non-invasive ventilation days (*p* = 0.038). Multiple logistic regression analysis confirmed that hydrocortisone treatment was independently associated with a reduced risk of Grade II + BPD incidence (OR: 0.287, 95% CI: 0.084–0.980).

**Conclusions:**

These findings suggest that early administration of low-dose hydrocortisone is associated with a reduced risk of Grade II^+^ BPD in extremely preterm infants born before 26 weeks of gestation.

## Introduction

Bronchopulmonary dysplasia (BPD) is a common complication among preterm infants, often leading to significant neurodevelopmental impairments (1) and abnormal lung function (2). Despite advancements in perinatal care and improved survival rates for extremely premature infants, the incidence of BPD has remained unchanged (3). Preterm infants who develop more severe forms of BPD are typically born at earlier gestational ages, have lower birth weights, and are more likely to be small for their gestational age. Data from the Vermont Oxford Network (VON) show that the incidence of Grade I or II BPD are 51.8% at 25 weeks' gestation, 48.5% at 26 weeks' gestation, and 38.1% at 27 weeks' gestation. Additionally, the incidence of BPD Grade III at these gestational ages was 7.1% at 25 weeks, 4.1% at 26 weeks and 2.8% at 27 weeks (4). These findings highlight that infants born before 26 weeks are at a significantly higher likelihood of developing BPD.

Lower cortisol level during the first week of life are correlated with increased lung inflammation and a higher likelihood of development of BPD in very low birth weight infants (5). While many factors contribute to the development of BPD, lung inflammation is believed to play a key role in both its onset and progression (6). Corticosteroids, as potent anti-inflammatory agents, can mitigate the risk of BPD by suppressing the inflammatory responses in preterm infants (7). Dexamethasone, a long-acting glucocorticoid, has been widely used for the prevention and treatment of BPD (8, 9). Its benefits include reducing the need for mechanical ventilation, decreasing the incidence of BPD at 28 days and 36 weeks postmenstrual age (PMA), and lowering neonatal mortality. However, due to concerns about long-term adverse effects-particularly cognitive impairment and cerebral palsy, dexamethasone is now reserved for infants who cannot be weaned from invasive ventilation more than 7 days of life (8).

Hydrocortisone, as an alternative corticosteroid, is considered a promising option for preventing BPD due to its no significant long-term negative neurological effects compared to other corticosteroids (10). Studies have shown that systemic hydrocortisone is not associated with a reduction in total brain tissue volume or an increased risk of cerebral palsy in infants (11, 12). The PREMILOC trial demonstrated that a low dose of hydrocortisone (8.5 mg) administered shortly after birth improved survival without BPD in extremely premature infants (13). In contrast, a higher dose (72.5 mg) initiated between postnatal days 7 and 14 did not improve BPD-free survival in infants with a gestational age of less than 30 weeks (14). These conflicting results may be explained by differences in dosage, timing of treatment initiation, and patient populations, underscoring the importance of early intervention in the most vulnerable infants to effectively prevent BPD.

Although hydrocortisone treatment is widely used in neonatal units globally, the optimal dosage, timing of initiation, and identification of the most suitable patient population remain unclear. These knowledge gaps highlight the need for further research to refine clinical practices and improve outcomes. To address these uncertainties, this study aims to evaluate the effectiveness of early low-dose hydrocortisone in preventing BPD among extremely premature infants born at less than 26 weeks of gestation.

## Materials and methods

### Patients and data collection

This retrospective case-control study was conducted in the Neonatal Intensive Care Unit (NICU) of Shenzhen Maternity and Child Healthcare Hospital from 2020 to 2023. Eligibility for the study included all preterm infants born with a gestational age (GA) of less than 26 weeks. Infants were excluded if they died within 14 days after birth or had major congenital heart malformations. Outcomes of the current intervention cohort were compared to historical controls who did not receive hydrocortisone therapy. After applying the inclusion and exclusion criteria, 33 extremely preterm infants treated with hydrocortisone within 24 h after birth and 33 GA-matched controls [matched by propensity score matching (PSM)] were included in the analysis. The study was approved by the Institutional Ethical Committee of Shenzhen Maternity and Child Healthcare Hospital, and the requirement for informed consent was waived by the committee [SFYLS(2024)-008].

Neonatal data were collected from the electronic medical records, including antenatal steroid treatment, delivery methods, GA, birth weight (BW), Apgar scores at 1 and 5 min, sex, and the duration of invasive mechanical ventilation, hemodynamically significant patent ductus arteriosus (HsPDA), intraventricular hemorrhage (IVH), Death.

The infants were divided into two groups: hydrocortisone and non-hydrocortisone, based on whether they received hydrocortisone treatment after birth. At our center, this approach was taken following a randomized study (13) and based on the physiologic levels of serum cortisol after birth, we adjusted the doses of hydrocortisone to address the insufficiency in extremely preterm infants. The hydrocortisone group received hydrocortisone sodium succinate at a dosage of 1 mg/kg per day, divided into 4 doses, for the first 3 days. This was followed by a reduced dosage of 0.75 mg/kg per day, administered in 3 doses, for the next 2 days. The frequency of dosing was then decremented by 1 dose every 2 days. The cumulative dosage of this treatment regimen is 6 mg/kg over a 9-day period. No other glucocorticoids were allowed during the 9-day treatment period.

The definition of BPD is now frequently graded according to the level of respiratory support needed at 36 weeks postmenstrual age (PMA), irrespective of the use or level of oxygen therapy (15). Diagnoses of IVH, hsPDA, and chorioamnionitis were as previously reported (16–18).

PSM based on GA and BW was utilized to identify controls for infants treated with hydrocortisone, with a match ratio of 1:1 and a match tolerance of 0.1. Data are presented as median (interquartile range) or frequency. Continuous variables were compared using Mann–Whitney *U* test and proportions using *χ*^2^ or Fisher's exact test. Potential risk factors for BPD were first evaluated in a univariable regression model (binary regression). Factors such as gestational age, birth weight, Apgar score at 1 min, surfactant use, and early hydrocortisone therapy were then entered into a multivariable logistic regression model to evaluate their independent contributions to the development of BPD. Two-sided *p*-values less than 0.05 were considered statistically significant. Odds ratios (OR) and 95% confidence intervals (CI) were calculated using both univariable and multivariate regression analysis. All statistical analyses were conducted using SPSS statistical software, version 23.0 (IBM Corporation).

## Results

### Case selection and characteristics of study subjects by groups

**Figure 1:**
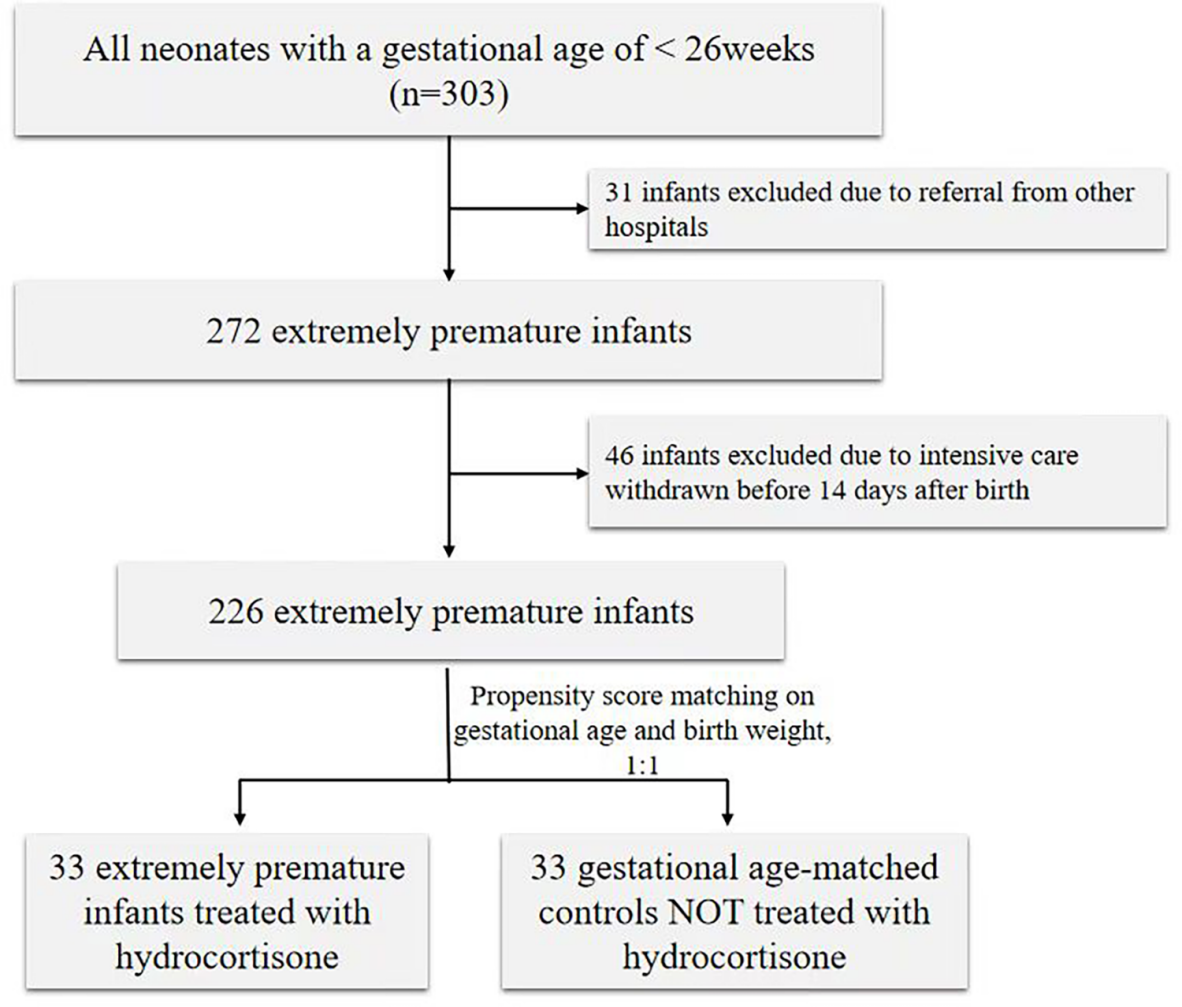
Flowchart of participant inclusion and exclusion.

During the study period, 303 preterm infants were screened with a GA below 26 weeks. Thirty-one infants were excluded due to referral from other hospitals, and 46 infants were excluded due to intensive care being withdrawn before 14 days after birth. Consequently, 33 preterm infants treated with hydrocortisone were matched with 33 preterm infants without hydrocortisone and included in the analysis (shown in Figure 1). The clinical characteristics of preterm infants with or without hydrocortisone treatment are summarized in Table 1. Apgar at 1st minute showed significant differences between the hydrocortisone group and the non-hydrocortisone group (Table 1). No significant differences were found in BW, GA, male sex, cesarean delivery, Apgar at 5st, chorioamnionitis or antenatal steroid use (Table 1).

**Table 1:** Clinical characteristics with and without early hydrocortisone.

Characteristics	No early hydrocortisone (*n* = 33)	Early hydrocortisone (*n* = 33)	*p* value
Gestational age (weeks, mean ± SD)	24.75 ± 0.80	24.78 ± 0.85	0.882
Birth weight (grams, mean ± SD)	747 ± 101	698 ± 102	0.057
Males (*N*, %)	22 (66.7)	20 (60.6)	0.609
Apgar at 1 min (median, IQR)	5 (3)	5 (4)	0.017
Apgar at 5 min (median, IQR)	8 (2)	8 (3)	0.052
Caesarean delivery (*N*, %)	5 (15.2)	10 (30.3)	0.142
Antenatal steroids (*N*, %)	22 (66.7)	24 (72.7)	0.592
Surfactant (*N*, %)	21 (63.6)	32 (96.9)	0.001
Chorioamnionitis (*N*, %)	11 (33)	12 (36.4)	0.796

IQR, interquartile range.

### Effects of treatment on the respiratory outcome

We found that the incidence of Grade II^+^ BPD was significantly lower in infants with hydrocortisone treatment (27.2%) compared with those without treatment (54.5%, *p* = 0.024) (Table 2). Meantime, hydrocortisone reduce the days of non-invasive ventilation (43 vs. 30 days, *p* = 0.038). However, hydrocortisone failed to reduce the time of supplemental oxygen, invasive ventilation and hospital stay days. Moreover, we found no significant difference in the overall incidence of BPD between the two groups (31 vs. 28, *p* = 0.427) (Table 2).

**Table 2:** Respiratory outcomes of infants with and without early hydrocortisone.

Outcomes	No early hydrocortisone (*n* = 33)	Early hydrocortisone (*n* = 33)	*p* value
Overall prevalence of BPD (*N*, %)	28 (84.8)	31 (93.9)	0.427
Grade Ⅱ^+^ BPD (*N*, %)	18 (54.5)	9 (27.2)	0.024
Grade Ⅲ BPD (*N*, %)	10 (30.3)	9 (27.3)	0.786
Invasive ventilation [days, median (IQR)]	22 (29)	22 (27)	0.484
Non-invasive ventilation [days, median (IQR)]	43 (30)	30 (21)	0.038
Supplemental oxygen [days, median (IQR)]	91 (44)	90 (42)	0.888
Hospital stay [days, median (IQR)]	99 (36)	98 (24)	0.773

IQR, interquartile range; BPD, bronchopulmonary dysplasia.

### The effect of treatment on secondary outcomes

Although nonsteroidal anti-inflammatory drug (NSAID) use for promoting PDA closure differed between the two groups, there was no significant difference in spontaneous intestinal perforation rates between the groups (Table 3). No differences were found in the incidence of IVH and HsPDA between the two groups. Of the four deaths in the hydrocortisone group, three discharge against medical advice. The last one died of early sepsis and circulatory instability. In the non-hydrocortisone group, there were two deaths, one discharged due to socioeconomic reasons and the other died of late-onset sepsis.

**Table 3:** The effect of treatment on secondary outcomes.

Outcomes	No early hydrocortisone (*n* = 33)	Early hydrocortisone (*n* = 33)	*p* value
HsPDA (*N*, %)	19 (57.6)	18 (54.5)	0.804
NSAIDs use (*N*, %)	22 (66.7)	14 (42.4)	0.048
SIP (*N*, %)	2 (6.1)	5 (15.2)	0.427
Late-onset sepsis (*N*, %)	3 (9.1)	1 (3)	0.613
Severe IVH (*N*, %)	4 (12.1)	5 (15.2)	0.720
Death (*N*, %)	2 (6.1)	4 (12.1)	0.672

HsPDA, hemodynamically significant patent ductus arteriosus; NSAIDs, non-steroidal anti-inflammatory drugs; SIP, spontaneous intestinal perforation; IVH, intraventricular hemorrhage; severe IVH, grade Ⅲ or Ⅳ.

**Table 4:** Multiple logistic regression analysis of selected variables associated with grade Ⅱ^+^ BPD.

Variables	Non-Grade Ⅱ^+^ BPD (*N* = 39)	Grade Ⅱ^+^ BPD (*N* = 27)	β/OR (95% CI)	*p* value
Gestational age (weeks, mean ± SD)	24.9 ± 0.8	24.6 ± 0.9	0.519 (0.211, 1.274)	0.152
Birth weight (grams, mean ± SD)	721 ± 98	725 ± 112	1.002 (0.995, 1.010)	0.510
Apgar at 1 min (median, IQR)	5 (3)	5 (1)	0.807 (0.624, 1.044)	0.103
Surfactant (*N*, %)	33 (84.6)	20 (74.1)	0.584 (0.124, 2.751)	0.496
Early hydrocortisone (*N*, %)	24 (61.5)	9 (33.3)	0.287 (0.084, 0.980)	0.046

OR, odds ratio; CI, confidence interval.

### Multiple logistic regression analysis of Grade II^+^ BPD

The univariable analysis showed that lower incidence of Grade II^+^ BPD was associated with Apgar at 1st and surfactant. After adjusting for known confounders including gestational age, birth weight, Apgar at 1st minute and surfactant, early hydrocortisone was independently associated with a lower risk of Grade II^+^ BPD (OR: 0.287, 95% CI: 0.084–0.980, *p* = 0.046) (Table 4).

## Discussion

This retrospective case-control study suggests that early administration of hydrocortisone, initiated shortly after extremely preterm birth, is associated with a reduce incidence of Grade II^+^ BPD at 36 weeks PMA. Additionally, we found that hydrocortisone treatment shortened the duration of non-invasive ventilation compared to the non-hydrocortisone group.

To our knowledge, this is the first retrospective analysis of early hydrocortisone use in preterm infants born at less than 26 weeks, and the cumulative dose of hydrocortisone tested here is the lowest ever evaluated for this purpose in extremely preterm neonates. Hydrocortisone has been used as a prophylactic treatment initiated in the first few days of life to prevent BPD (13, 19, 20). These trials demonstrated a significant increase in the rate of survival without BPD at 36 weeks of PMA, which aligns partially with our findings. Moreover, another individual patient data Meta-analysis showed that early low-dose hydrocortisone therapy is beneficial for survival without BPD in very preterm infants (21). However, a randomized clinical trial of hydrocortisone initiated 7 days after birth in preterm infants under 30 weeks' GA failed to demonstrate a reduction in BPD incidence (14). In addition, a retrospective study indicated that hydrocortisone administered for respiration deterioration did not prevent BPD (22).

Meta-analyses have shown that early hydrocortisone treatment may significantly reduce mortality, though it does not appear to impact the incidence of BPD (23). We speculate that these discrepancies in findings regarding hydrocortisone's effects on BPD and mortality could be due to variations in the timing of hydrocortisone administration and differences in the patient populations studied. In our study, hydrocortisone treatment reduced the duration of non-invasive ventilation, which may help lower the incidence of Grade II + BPD. We hypothesize that early hydrocortisone administration in preterm infants could stabilize circulation, reduce ventilator parameters, and mitigate lung inflammation, potentially leading to a shorter duration of ventilator dependence.

Our results indicated no significant difference in the number of days requiring invasive ventilation between the hydrocortisone-treated group and the control group. This finding aligns with previous reports suggesting that hydrocortisone administration within 36 h of birth does not reduce the duration of invasive ventilation (24). However, our finding contrasts with the PREMILOC trial (13), which reported that early hydrocortisone administration facilitates tracheal extubation by the end of treatment. We hypothesize that this discrepancy may be attributed to the unique characteristics of our study population, consisting exclusively of infants born at less than 26 weeks' gestational age. These extremely premature infants often experience severe lung immaturity and injury (15, 22), which may prolong the time needed to wean from invasive ventilation. While hydrocortisone may aid in promoting lung maturation, the extent of its effect might not be sufficient to offset the significant challenges posed by such extreme prematurity. The potential mechanisms by which hydrocortisone facilitates lung maturation and recovery in immature lungs remain unclear. Hydrocortisone is thought to modulate inflammation (25), enhance surfactant production, and promote alveolar development (26), but further research is needed to elucidate these pathways in detail.

The generalizability of the study is limited by its small sample size and retrospective design, which preclude definitive causal inferences regarding treatment effects. Additionally, cortisol levels were not measured before or after hydrocortisone administration, limiting insights into physiological responses. Despite these limitations, our findings provide valuable evidence, emphasizing the need for larger, prospective studies to confirm the benefits and address remaining uncertainties about early low-dose hydrocortisone in extremely preterm infants.

## Conclusion

In summary, our case-control study demonstrated that early low-dose hydrocortisone significantly reduced the incidence of Grade II^+^ BPD in infants born at less than 26 weeks' gestation. While these findings highlight the potential of this treatment strategy, further validation through large-scale, randomized controlled trials is essential to confirm its efficacy and safety, as well as to establish optimal dosing and long-term outcomes.

## Data Availability

The original contributions presented in the study are included in the article/Supplementary Material, further inquiries can be directed to the corresponding authors.
